# *Wolbachia* enhances insect‐specific flavivirus infection in *Aedes aegypti* mosquitoes

**DOI:** 10.1002/ece3.4066

**Published:** 2018-05-08

**Authors:** Hilaria E. Amuzu, Kirill Tsyganov, Cassandra Koh, Rosemarie I. Herbert, David R. Powell, Elizabeth A. McGraw

**Affiliations:** ^1^ School of Biological Sciences Monash University Clayton Vic. Australia; ^2^ Monash Bioinformatics Platform Monash University Clayton Vic. Australia; ^3^ Department of Entomology Pennsylvania State University University Park Pennsylvania

**Keywords:** biocontrol, dengue, flavivirus, insect‐specific flavivirus, mosquitoes, virome, *Wolbachia*

## Abstract

Mosquitoes transmit a diverse group of human flaviviruses including West Nile, dengue, yellow fever, and Zika viruses. Mosquitoes are also naturally infected with insect‐specific flaviviruses (ISFs), a subgroup of the family not capable of infecting vertebrates. Although ISFs are not medically important, they are capable of altering the mosquito's susceptibility to flaviviruses and may alter host fitness. *Wolbachia* is an endosymbiotic bacterium of insects that when present in mosquitoes limits the replication of co‐infecting pathogens, including flaviviruses. Artificially created *Wolbachia*‐infected *Aedes aegypti* mosquitoes are being released into the wild in a series of trials around the globe with the hope of interrupting dengue and Zika virus transmission from mosquitoes to humans. Our work investigated the effect of *Wolbachia* on ISF infection in wild‐caught *Ae. aegypti* mosquitoes from field release zones. All field mosquitoes were screened for the presence of ISFs using general degenerate flavivirus primers and their PCR amplicons sequenced. ISFs were found to be common and widely distributed in *Ae. aegypti* populations. Field mosquitoes consistently had higher ISF infection rates and viral loads compared to laboratory colony material indicating that environmental conditions may modulate ISF infection in *Ae. aegypti*. Surprisingly, higher ISF infection rates and loads were found in *Wolbachia*‐infected mosquitoes compared to the *Wolbachia‐*free mosquitoes. Our findings demonstrate that the symbiont is capable of manipulating the mosquito virome and that *Wolbachia*‐mediated viral inhibition is not universal for flaviviruses. This may have implications for the *Wolbachia‐*based DENV control strategy if ISFs confer fitness effects or alter mosquito susceptibility to other flaviviruses.

## 1 INTRODUCTION

Mosquitoes transmit a wide range of pathogens including viruses deemed arboviruses that cause widespread morbidity and mortality in humans and animals (Mackenzie, Gubler, & Petersen, 2004; Mackenzie et al., 1994). These viruses belong to diverse families including the *Flaviviridae* (genus: *Flavivirus*) that are positive single‐stranded RNA viruses (Fauquet, Mayo, Maniloff, Desselberger, & Ball, 2005; Karabatsos, 1978). Flaviviruses include Japanese encephalitis virus and Murray Valley encephalitis virus transmitted by *Culex* species (Erlanger, Weiss, Keiser, Utzinger, & Wiedenmayer, 2009; Kay, Fanning, & Carley, 1984), West Nile virus (WNV) transmitted by a diverse group of mosquitoes including *Aedes* and *Culex* species (Mackenzie et al., 2004) and yellow fever virus (YFV), Zika virus (ZIKV), and dengue virus (DENV) that are all transmitted by *Aedes aegypti* and *Aedes albopictus* (Black et al., 2002; Hall‐Mendelin et al., 2016; Hayes, 2009). Dengue fever caused by DENV is a severely debilitating disease with 40% of the world's population at risk of infection and an estimated 300 new infections reported yearly (Bhatt et al., 2013). A newly emerging threat to global health is Zika fever caused by the ZIKV with outbreaks reported in both tropical and subtropical regions (Pujhari & Rasgon, 2016; Younger, 2016).

Mosquitoes are also known to naturally harbor flaviviruses that are incapable of infecting humans or other vertebrate animals and are therefore known as insect‐specific flaviviruses (ISFs). Cell fusing agent virus was the first ISF to be discovered in *Ae. aegypti* in 1975 (Stollar & Thomas, 1975). Since then, several others have been described including *Culex flavivirus* (CxFv) in *Culex pipiens*, Kamiti River virus in *Ae. aegypti,* and Palm Creek virus in *Coquillettidia xanthogaster* mosquitoes (Crabtree, Sang, Stollar, Dunster, & Miller, 2003; Hobson‐Peters et al., 2013; Hoshino et al., 2007). Even though ISFs are not directly associated with disease in vertebrates, there is growing interest in their effect on co‐infecting arboviruses. In mosquito cell culture, some studies have shown that ISFs suppress flaviviruses, including WNV by CxFV and Murray Valley virus by Palm creek virus (Bolling, Olea‐Popelka, Eisen, Moore, & Blair, 2012; Hobson‐Peters et al., 2013). However, this effect, known as superinfection exclusion (Billecocq, Vazeille‐Falcoz, Rodhain, & Bouloy, 2000; Geib et al., 2003; Karpf, Lenches, Strauss, Strauss, & Brown, 1997; McAlister & Barrett, 1977; Nethe, Berkhout, & van der Kuyl, 2005; Pesko & Mores, 2009), was not observed in field mosquito populations where a positive association was found between WNV and CxFV. This suggests that the presence of CxFV may make mosquitoes more susceptible to WNV (Newman et al., 2011). Conflicting results were also observed in other studies where CxFV did not have an effect on replication, transmission, or dissemination of WNV in *Culex quinquefaciatus* (Kent, Crabtree, & Miller, 2010).

*Wolbachia pipientis* is an endosymbiotic bacterium of insects that is currently being developed as a biocontrol agent (Iturbe‐Ormaetxe, Walker, & LO'Neill, 2011; Moreira et al., 2009; Walker et al., 2011). *Wolbachia* is naturally present in about 52% of arthropods and is maternally inherited (Weinart, Arauju‐Jnr, Ahamed, & Welch, 2015). The symbiont manipulates host reproduction, such that the eggs of *Wolbachia*‐free females do not hatch when they have been fertilized by a *Wolbachia*‐infected male (Serbus, Casper‐Lindley, Landmann, & Sullivan, 2008). This phenomenon, referred to as cytoplasmic incompatibility (CI), leads to the spread and invasion of *Wolbachia* into wild populations. Another desirable phenotype of *Wolbachia* is its ability to inhibit infection of the host with other pathogens (Bian, Xu, Lu, Xie, & Xi, 2010; Bian et al., 2013; Frentiu et al., 2014; Walker et al., 2011). This “pathogen blocking” by *Wolbachia* was first observed in *Drosophila melanogaster* (Hedges, Brownlie, O'Neill, & Johnson, 2008; Teixeira, Ferreira, & Ashburner, 2008) where flies infected with *Drosophila* C virus (DCV) and cricket paralysis virus and accumulated virus at a slower rate leading to higher survival rates compared to *Wolbachia‐*free controls (Hedges et al., 2008).

While present in 28% of mosquitoes such as *Ae. albopictus, Cx. pipiens,* and *Cx. quinquefaciatus, Wolbachia* was not thought to be present in the malaria vectors (*Anopheles* species) or the primary vector of DENV (*Ae. aegypti*) (Kittayapong, Baisley, Baimai, & O'Neill, 2000). There have been recent reports however of sporadic infections in *Anopheles coluzzii* (Shaw et al., 2016) and *Anopheles gambiae* (Gomes et al., 2017) and in a single population of *Ae. aegypti* (Coon, Brown, & Strand, 2016). Over the last decade three different *Wolbachia* strains have been artificially introduced into *Ae. aegypti* where they form stably inherited infections. These are *w*MelPop‐CLA and *w*Mel from *Drosophila* (McMeniman et al., 2009; Walker et al., 2011), *w*AlbB from *Ae. albopictus* (Xi, Dean, Khoo, & Dobson, 2005), and *w*Mel*w*AlbB (Joubert et al., 2016) that is a superinfection of *w*Mel and *w*AlbB. *Wolbachia*‐mediated pathogen blocking has now been observed for arboviruses such as WNV (Glaser & Meola, 2010), YFV (van den Hurk et al., 2012), DENV (Bian et al., 2010; Frentiu, Robinson, Young, McGraw, & O'Neill, 2010; Moreira et al., 2009; Walker et al., 2011), ZIKV (Aliota, Peinado, Velez, & Osorio, 2016; Dutra et al., 2016), and Chikungunya virus (van den Hurk et al., 2012; Moreira et al., 2009). *Wolbachia* is currently being released into populations of *Ae. aegypti* globally to test whether it may be effective at limiting DENV and ZIKV transmission to humans (Ritchie, 2014) (see http://www.eliminatedengue.com). The first releases in Australia demonstrated that *Wolbachia* was able to invade wild *Ae. aegypti* populations and remain at near 100% frequency (Hoffmann et al., 2011). Subsequent releases in DENV endemic regions are being used to test for efficacy of human infection control (Ritchie, 2014).

Although largely consistent, there are some reports of *Wolbachia* enhancing rather than preventing pathogen co‐infection including *Plasmodium* and WNV within *Anopheles gambiae* and *Culex tarsalis*, respectively (Dodson et al., 2014; Hughes, Vega‐Rodriguez, Xue, & Rasgon, 2012). In both of these instances, however, the mosquitoes were only transiently infected with *Wolbachia* via artificial micro‐injection and so may not be representative of insects with germline tissue infections (Joubert & O'Neill, 2017). Several vectors naturally infected with *Wolbachia* have also exhibited increased susceptibility to pathogens. This has been shown in *Cx. pipiens* and *Spodoptera exempta* (African armyworm moth) with increased susceptibility to *Plasmodium* and nucleopolyhedrovirus (double‐stranded DNA virus), respectively (Graham, Grzywacz, Mushobozi, & Wilson, 2012; Zele et al., 2014). In contrast, the natively infected *Ae. albopictus* exhibits reduced susceptibility and transmission of DENV (Mousson et al., 2012). These studies suggest that *Wolbachia*‐mediated pathogen blocking may depend on several factors that are influenced by specific *Wolbachia* strain and pathogen–host interactions including history of association.

The mechanistic basis of *Wolbachia*‐mediated pathogen blocking is still not well understood (Terradas & McGraw, 2017). Currently, pathogen blocking has been partly attributed to the ability of *Wolbachia* to increase the innate immune responses of the host, thereby making it resist subsequent pathogen infection (Bian et al., 2010; Pan et al., 2012; Rances, Ye, Woolfit, McGraw, & O'Neill, 2012). It has also been hypothesized that competition between *Wolbachia* and pathogens for key host resources such as lipids (Caragata et al., 2013) and intracellular space (Moreira et al., 2009) may underpin blocking. This may be particularly relevant for viruses that require lipids for attachment and entry into host cells and for replication (Lu, Cassese, & Kielian, 1999; Mackenzie, Khromykh, & Parton, 2007). Most recently, there is some evidence primarily from *Drosophila* that *Wolbachia* may be modifying host cellular structures or organelles rendering them less hospitable to viral replication (Rainey et al., 2016; White et al., 2017). A range of studies also point to a correlation between *Wolbachia* densities and the strength of blocking (Frentiu et al., 2010; Lu, Bian, Pan, & Xi, 2012; Osborne, Iturbe‐Ormaetxe, Brownlie, O'Neill, & Johnson, 2012), a trend that would be expected with any of the above explanations for blocking.

While *Wolbachia* appears to shift the composition of the microbiome in mosquitoes (Audsley, Seleznev, Joubert, O'Neill, & McGraw, 2018), little is known about its effects on ISFs. There is also little known about the effects of ISFs on mosquito health. If these infections affect survival or reproduction, *Wolbachia*‐infected insects in the field may receive an advantage in carrying the symbiont. For example, native viruses in *Drosophila* such as DCV and cricket paralysis virus reduce host fitness and *Wolbachia* infections are hence beneficial (Hedges et al., 2008). A survey in wild populations of *D. melanogaster* demonstrated that *Wolbachia* infection was not associated with changes in the diversity of native viruses in the insect (Webster et al., 2015). *Wolbachia* infections in *Ae. aegypti* differ significantly from those found in *D. melanogaster* however, exhibiting higher symbiont loads (McGraw, Merritt, Droller, & O'Neill, 2002; McMeniman et al., 2009; Moreira et al., 2009; Walker et al., 2011), broader tissue distributions (Moreira et al., 2009; Walker et al., 2011), greater activation of the immune response (McGraw et al., 2002; McMeniman et al., 2009; Moreira et al., 2009; Walker et al., 2011), and greater fitness costs (McMeniman, Hughes, & O'Neill, 2011; Min & Benzer, 1997). These discrepancies may result from different periods of association/evolutionary history, long (~5,000 years) in the case of *D. melanogaster* (Richardson et al., 2012) and short (<10 years) in the case of the newly infected *Ae. aegypti* (Walker et al., 2011). Understanding the fitness consequences of *Wolbachia* for *Ae. aegypti* is necessary to effectively model the long‐term stability and success of the symbiont as a biocontrol agent in wild populations.

Our work focused on determining if *Wolbachia*‐mediated viral blocking extends to naturally occurring flaviviruses in mosquitoes. We sampled *Wolbachia*‐infected mosquitoes from field release sites in Cairns, Australia, and symbiont‐free mosquitoes from nearby control areas outside of the *Wolbachia* release zone. Using flavivirus general degenerate primers, we amplified the NS5 region of the virus genome and sequenced the PCR amplicons of individual positive mosquitoes using Miseq Illumina sequencing. We further screened laboratory colonies and field mosquitoes using primers designed specifically for several of the ISF sequences. We found that ISFs are common and widely distributed in *Ae. aegypti* populations with infection rates and abundance consistently higher in field mosquitoes compared to the laboratory colonies. This possibly indicates that variations in environmental conditions could be playing a role in controlling ISF infection in *Ae. aegypti*. Unexpectedly, we found that *Wolbachia* enhanced ISF infection rates and loads in *Ae. aegypti* demonstrating that the antivirus effect associated with *Wolbachia* is not common to all flaviviruses. These findings may have implications for *Wolbachia*‐DENV control if ISFs affect host fitness or play a role in mosquito susceptibility to flaviviruses.

## 2 MATERIALS AND METHODS

### 2.1 Mosquito sampling

*Wolbachia‐*infected and uninfected (wild‐type) *Ae**. **aegypti* mosquitoes were sampled from three different communities in Cairns, Australia. The *Wolbachia*‐infected mosquitoes were sampled in 2013 from two sites where *w*Mel mosquitoes were released in 2011 (Hoffmann et al., 2011) and 2013 (Ritchie, 2014). These are Gordonvale (GV) and Parramatta Park (PP), respectively. Wild‐type mosquitoes were sampled from Holloways Beach (HB) that is outside the original release zone. BG‐sentinel mosquito traps (Biogen, Germany) were set randomly in these areas, and adult mosquitoes were collected overnight. *Ae**. **aegypti* mosquitoes were morphologically identified and placed in vials containing 80% ethanol. A total of 95 individual mosquitoes were assessed across the three collection sites (39 from GV, 21 from PP, and 35 from HB) for the presence of ISFs.

### 2.2 Screening for insect‐specific flaviviruses

RNA and DNA were simultaneously extracted from each mosquito using the TRIzol^®^ method from Invitrogen (Life technologies, Carlsbad, CA, USA). The DNA was used to screen for the presence of *Wolbachia* infection via qPCR as previously described (Frentiu et al., 2014). Two samples each from PP and GV were found to be *Wolbachia* negative and were excluded from further analysis. All HB samples were confirmed to be *Wolbachia* negative as expected. The RNA was DNase‐treated to remove genomic DNA contamination using DNase 1 recombinant RNase‐free (Roche, Germany). Reverse transcription of RNA to cDNA and the PCR amplification of the NS5 region using general degenerate flavivirus primers were carried out following the protocol of Sanchez‐Seco et al. (2005). Briefly, reverse transcription of RNA to cDNA and subsequent first‐round amplification were carried out using 1 μg of RNA in the Access RT‐PCR System (Promega, Madison, WI, USA). Reverse transcription controls that did not include enzyme were included in each run to rule out genomic DNA contamination. One microliter of the first‐round amplification was then used for the second round of nested PCR. All PCR products were run in a C1000™Thermal Cycler (Bio‐Rad, CA, USA). PCR products (143 bp) were analyzed using gel electrophoresis on a 2% Agarose gel (Sigma, Life Science, USA) stained with RedSafe^™^ (iNtRON Biotechnology). Products were then visualized on the Quantum gel documentation system (Fisher Biotec).

### 2.3 Sequencing of PCR products

A subset of six individual mosquitoes from each of the three sites were selected for further processing for sequencing. To ensure a good representation of an area, the samples were selected from different traps that were not in close proximity. One microliter of the second‐round PCR product of each of the selected individual samples was used as the template for a 5‐cycle amplification with primers barcoded with Illumina sequence adapters (Berry, Ben Mahfoudh, Wagner, & Loy, 2011). The PCR amplicons were analyzed using gel electrophoresis as described above. The amplicons were then excised and gel extracted using QIAquick gel extraction kit (Qiagen, Germany) following manufactures instructions. Extracted samples were then paired‐end sequenced using MiSeq at Ramaciotti sequencing center, NSW, Australia.

### 2.4 Processing and clustering of sequences

All sequences were processed with cutadapt (Martin, 2011) as the very first step, to remove degenerate primers used for the PCR amplicons. Cutadapt was run with the following settings; minimum overlap 10 nucleotides, minimum read length: 1 nucleotide (this is mainly to allow downstream R1 and R2 merging), and number of attempts to trim a primer was set to 2. To classify sequences into Operational Taxonomical Units (OTUs), vsearch (Rognes, Flouri, Nichols, Quince, & Mahe, 2016) was used to merge reads with number of mismatches set to two nucleotides, the number of allowed N's set to 0, and the minimum overlap set to 32 bases. This was followed by filtering reads based on expected error of 1. Identical sequences were then collapsed into a single sequence (dereplication) and then clustered using 97% identity. Contingency table of cluster counts was subsequently generated using usearch (Edgar, 2010). The OTUs were finally imported into the flavivirus Database under The Virus Pathogen Resource (http://www.viprbrc.org) and the Basic Local Alignment Search Tool (BLAST) used to find the closest match or hit of each OTU.

### 2.5 Phylogenetic reconstruction

Individual OTUs were aligned with their best hits using the multiple sequence comparison by log‐expectation (MUSCLE) tool (Edgar, 2004) provided by The European Bioinformatics Institute (EMBL‐EBI). The aligned sequences were manually trimmed and then imported into Phylogeny.fr together with other common flaviviruses (Dereeper, Audic, Claverie, & Blanc, 2010; Dereeper et al., 2008). The one click mode of Phylogeny.fr that uses MUSCLE for sequence alignment and maximum likelihood (PhyML) for tree building with aLRT (approximate likelihood‐ratio test) statistical test for branch support values, and TreeDyn for tree drawing was used for the phylogeny tree (Anisimova & Gascuel, 2006; Chevenet, Brun, Banuls, Jacq, & Christen, 2006; Guindon & Gascuel, 2003).

### 2.6 Screening of field mosquitoes using OTU‐specific primers

Primers (Table 1) were designed for 7 ISF OTUs that were selected based on their abundance and diversity in the sequenced samples as well as their phylogenetic positions. All primers were designed using the Primer3 tool in The Virus Pathogen Resource database (viprbrc.org). Quantitative PCR using SYBR Green (Roche, Applied Science, Switzerland) in a LightCycler480 (Roche, Applied Science, Switzerland) was then used to validate the presence and abundance of OTUs in all mosquitoes sampled from the field. This was performed using 1 μl of the first‐round amplification, 5 μl of 5X SYBR Green master mix, and 0.5 μl of 10 mmol/L each of forward and reverse primers in a total volume of 10 μl. The cycling conditions were pre‐incubation at 95°C for 5 min, 45 amplification cycles of 95°C for 10 s, 60°C for 10 s, and 72°C for 10 s followed by a melting curve at 95°C for 5 s, 65°C for 1 min, and a continuous acquisition mode at 97°C. The housekeeping gene RPS17 (Cook et al., 2006; Thellin et al., 1999) was used to normalize virus abundance.

**Table 1:** OTU‐specific primer pairs used for PCR amplification

OTUs	Forward primer	Reverse primer
OTU1	AGAAGCAACCGACCATAGCT	CCAGATATCGACTTCCCAGCC
OTU2	AGAAGGAGAAAAAGCCCAGCC	GCTAGAGCCTCAAATTCAAGGA
OTU3	TAGCTGGGGAGCCGAAAG	GGCCTCATATTCCAGATATCGACT
OTU16	GTGTGCACAACATGATGGGG	TTGAGGAAGCCCAATGGTCC
OTU20	TCAACACGGACCACTGGAAG	TGTTGAGAAAGCCCATGGTGT
OTU21	TTCCTCAACACGGACCAGTG	GTGGTCTTGTAGAGAAGCCCC
OTU25	GCCACTGGGAGCATTAACCT	GTCCGTGTTAGAAAGCCCCA

### 2.7 Screening of laboratory mosquitoes

*Wolbachia*‐infected and wild‐type lines maintained in the laboratory were screened for ISFs to ascertain whether they showed similar patterns as seen in field‐caught mosquitoes. The *Wolbachia*‐infected mosquitoes were previously sampled from field release sites in Cairns, Australia (Hoffmann et al., 2011), and the wild‐type mosquitoes was from Babinda, Australia (outside the release zone). To avoid genetic drift between the two lines, 20% of wild‐type males were outcrossed with the *Wolbachia*‐infected females at every generation. Mosquitoes were maintained only on 10% sucrose, and 4‐ to 7‐day‐old females were used for this study. RNA was extracted from 59 and 56 individual mosquitoes each from the *Wolbachia*‐infected and the wild‐type populations, respectively. The RNA extraction and DNase treatment were carried out as above. Using random primers (125 ng/μl), the first‐strand cDNA synthesis was carried out with SuperScript III reverse transcriptase (Invitrogen, California USA) following the manufacturer's instructions. The cDNA synthesis was run in a C1000™ Thermal Cycler (Bio‐Rad, California USA). Quantitative PCR using SYBR Green (Roche, Applied Science, Switzerland) was then carried out in a LightCycler480 (Roche, Applied Science, Switzerland) using 1.5 μl of cDNA, 5 μl of 5X SYBR Green master mix, and 0.5 μl of 10 mmol/L each of forward and reverse OTU‐specific primers in a total volume of 10 μl. The cycling conditions were as above.

### 2.8 Statistical analysis

To determine whether there was an association between *Wolbachia* infection and the presence/absence of ISFs, a binary logistic regression was carried out with presence/absence of ISFs as a dependent variable and *Wolbachia* infection status as a predictor in a generalized linear model to analyze the following: (1) infection rates in all the field mosquitoes, (2) infection rates in the sequenced mosquitoes, (3) infection rates in the field mosquitoes after RT‐qPCR, and (4) infection rates in the laboratory samples. These analyses were performed in SPSS^®^ (IBM Statistics for Windows, Version 20.0). Where multiple models were run for individual OTUs, we utilized a Bonferroni multiple test correction. A Mann–Whitney test in GraphPad Prism (version 6) was used to analyze differences in ISF abundance between the wild‐type and *w*Mel mosquitoes.

## 3 RESULTS

### 3.1 *Wolbachia* infection is associated with higher rates of ISF infection as measured by ISF generalist primers in PCR

We observed high ISF infection rates in the all field‐collected samples as measured by PCR; 100% and 95% for GV (Gordonvale) and PP (Parramatta Park), respectively, and 74% for HB (Holloways Beach). If we test for the effect of *Wolbachia* in dictating infection frequency, we see that it is significant (Wald = 7.80; *df* = 1; *p* = .005). Our findings suggest that ISFs are a common feature of the mosquito virome and that *Wolbachia* may be enhancing the frequency of infection in the field.

### 3.2 The ISFs include well‐characterized viruses as well as what appear to be novel viruses

**Figure 1:**
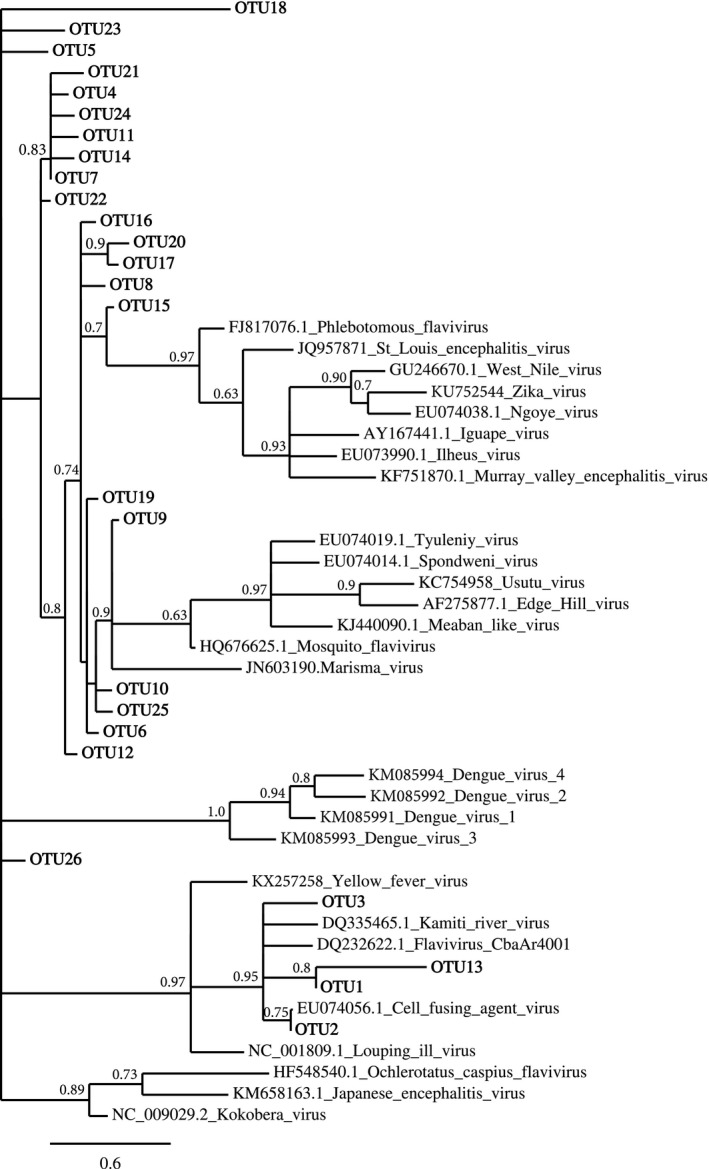
Maximum likelihood tree for the 26 ISF OTUs, their best reference hits, and key flaviviruses. Numbers at the nodes depict branch support values

To identify specific ISFs in the *Ae. aegypti* field populations, we sequenced a subset of samples that were ISF positive for both the wild‐type (*n* = 6) and *w*Mel (*n* = 12) mosquitoes. Following clustering analysis, a total of 26 “unique” ISF OTUs were identified in the sequenced samples (Figure 1). A list of all the OTUs and their sequences can be found in Table S1. Four OTUs (1, 2, 3, and 13) were very similar (>80%, Table 2) to the previously described ISFs Kamiti river virus, Cell fusing agent, and CbaAr4001. This group also forms a strongly supported (95%) phylogenetic cluster (Figure 1). OTU2's closest relative (75% bootstrap support) was Cell fusing agent. The majority of the OTUs, however, had low similarity (<50%) to known ISFs and OTUs 25 and 26, which had no match (Table 2). This lack of close relatives is recapitulated in the poor resolution within the phylogeny (Figure 1). There is some evidence of relatedness for OTU 9 that clusters with a group containing other ISFs including Mosquito_flavivirus and Marisma virus (90% support). The similarity of OTUs 4, 11, 14, 21, 22, 24 with one another suggest they may all be variants of a single virus. In summary, our results show evidence of several well‐characterized ISFs but also a large number of novel viruses in our *Ae. aegypti* population.

**Table 2:** OTUs with best hits/match reference

OTU	Best match name	Best match accession	Length of match	Bits score	e‐score	Identity	% Similarity
1	Kamiti River virus	DQ335465.1	124	178 (90)	5.00E‐44	106/110 (96%)	96.00
2	Cell fusing agent virus	EU074056.1	229	190 (96)	9.00E‐48	99/100 (99%)	99.00
3	Flavivirus CbaAr4001	DQ232622.1	87	155 (78)	5.00E‐37	84/86 (97%)	97.67
4	West Nile virus	GU246670.1	186	46.1 (23)	2.00E‐04	26/27 (96%)	57.81
5	Meaban‐like virus	KJ440090.1	124	48.1 (24)	6.00E‐05	24/24 (100%)	52.00
6	Dengue 2 virus	FJ392598.1	144	48.1 (24)	2.00E‐05	24/24 (100%)	43.04
7	Iguape virus	EU074054.1	229	48.1 (24)	6.00E‐05	24/24 (100%)	52.17
8	Tyuleniy virus	EU074019.1	232	48.1 (24)	1.00E‐04	24/24 (100)	52.92
9	Usutu virus	KC754958.1	10,745	44.1 (22)	1.00E‐03	22/22 (100%)	43.93
10	Spondweni virus	EU074014.1	232	44.1 (22)	1.00E‐03	25/26 (96%)	55.10
	West Nile virus	GU246670.1	186	44.1 (22)	1.00E‐03	25/26 (96%)	50.00
11	Spondweni virus	EU074014.1	232	44.1 (22)	1.00E‐03	25/26 (96%)	53.12
	West Nile virus	GU246670.1	186	44.1 (22)	1.00E‐03	25/26 (96%)	53.12
	St Louis encephalitis virus	JQ957871.1	2,718	44.1 (22)	1.00E‐03	25/26 (96%)	45.56
	Ilheus virus	EU073990.1	232	44.1 (22)	1.00E‐03	25/26 (96%)	46.94
12	Mosquito flavivirus	HQ676625.1	165	44.1 (22)	4.00E‐03	24/25 (96%)	44.87
	Phlebotomus flavivirus	FJ817076.1	157	42.1 (21)	4.00E‐03	24/25 (96%)	48.15
13	Mosquito flavivirus	HQ676625.1	165	42.1 (21)	4.00E‐03	24/25 (96%)	96.97
	Kamiti River virus	DQ335465.1	124	63.9 (32)	1.00E+09	32/32 (100%)	80.95
	Flavivirus CbaAr4001	DQ232622.1	87	63.9 (32)	1.00E‐09	32/32 (100%)	59.49
14	Mosquito flavivirus	HQ676625.1	165	42.(21)	4.00E‐03	24/25 (96%)	47.95
	Phlebotomus virus	FJ817076.1	157	42.1 (21)	4.00E‐03	24/25 (96%)	49.37
15	Louping ill‐like virus	NC_001809.1	10,871	40.1 (20)	2.80E‐02	20/20 (100%)	45.70
16	Japanese encephalitis virus	HQ223287.1	10,296	38.2 (19)	5.10E‐02	25/27 (92%)	50.72
	Edge Hill virus	AF275877.1	986	38.2 (19)	5.10E‐02	25/27 (92%)	50.72
17	Marisma virus	JN603190.1	1,008	44.1 (22)	1.00E‐03	22/24 (100%)	50.00
18	Kokobera virus	NC_009029.2	10,874	42.1 (21)	5.00E‐03	21/21 (100%)	42.39
	Tick‐borne encephalitis virus	KT224352.1	10,619	42.1 (21)	5.00E‐03	21/21 (100%)	45.75
	Dengue 2 virus	FJ392595.1	144	42.1 (21)	5.00E‐03	21/21 (100%)	37.11
19	Murray Valley encephalitis virus	KF751870.1	11,012	32.2 (16)	3.60E+00	19/20 (95%)	57.79
	St Louis encephalitis virus	JQ957871	2,718	32.2 (16)	3.60E+00	19/20 (95%)	49.35
	Dengue 2 virus	FJ392598.1	144	32.2 (16)	3.60E+00	19/20 (95%)	42.86
20	*Ochlerotatus caspius* flavivirus‐like virus	HF548540	9,839	34.2 (17)	8.80E‐01	17/17 (100%)	43.42
21	Hepatitis C virus	JQ060123.1	336	32.2 (16)	3.30E+00	16/16 (100%)	41.67
	Dengue 1 virus	M87512.1	10,717	32.2 (16)	3.30E+00	16/16 (100%)	52.11
	Usutu virus	NC_006551.1	11,066	32.2 (16)	3.30E+00	16/16 (100%)	45.33
	West Nile virus	KX547594.1	10,787	32.2 (16)	3.30E+00	16/16 (100%)	49.33
	Japanese encephalitis virus	KM658163.1	10,965	32.2 (16)	3.30E+00	16/16 (100%)	46.67
22	West Nile virus	GU246670.1	186	44.1 (22)	1.00E‐03	25/26 (96%)	61.8
	Ngoye virus	EU074038.1	232	44.1 (22)	1.00E‐03	25/26 (96%)	50.00
23	West Nile virus	JX041630.1	10,810	40.1 (20)	1.40E‐02	23/24 (95%)	50.00
	Meaban virus	KJ440090.1	124	40.1 (20)	1.40E‐02	23/24 (95%)	51.39
24	Meaban virus	KJ440090.1	124	40.1 (20)	1.50E‐02	20/20 (100)	53.33
	Iguape virus	AY167441.1	2,669	40.1 (20)	1.50E‐02	20/20 (100)	51.35
25	Unidentified flavivirus 1	–	–	–	–	–	–
26	Unidentified flavivirus 2	–	–	–	–	–	–

### 3.3 *Wolbachia* is often associated with higher ISF frequencies

**Figure 2:**
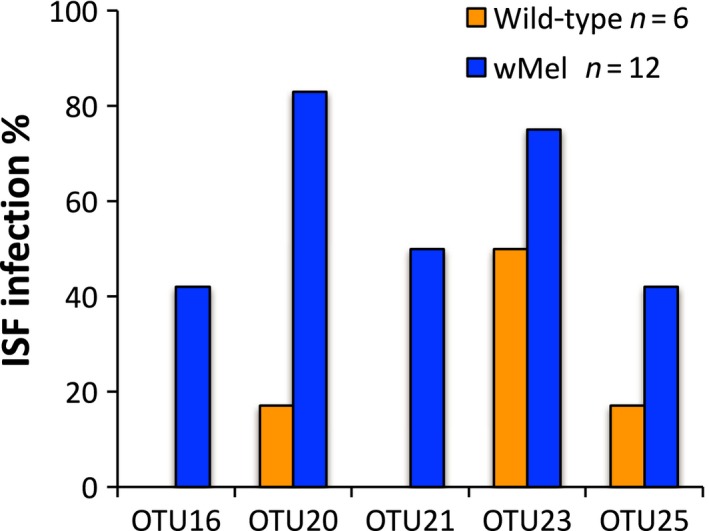
ISF infection rates for OTUs not fixed in both WT and *Wolbachia*‐infected mosquitoes from the field as determined by sequencing. Across these 5 OTUs, *w*Mel mosquitoes exhibited higher infection rates (*p* = .019)

**Figure 3:**
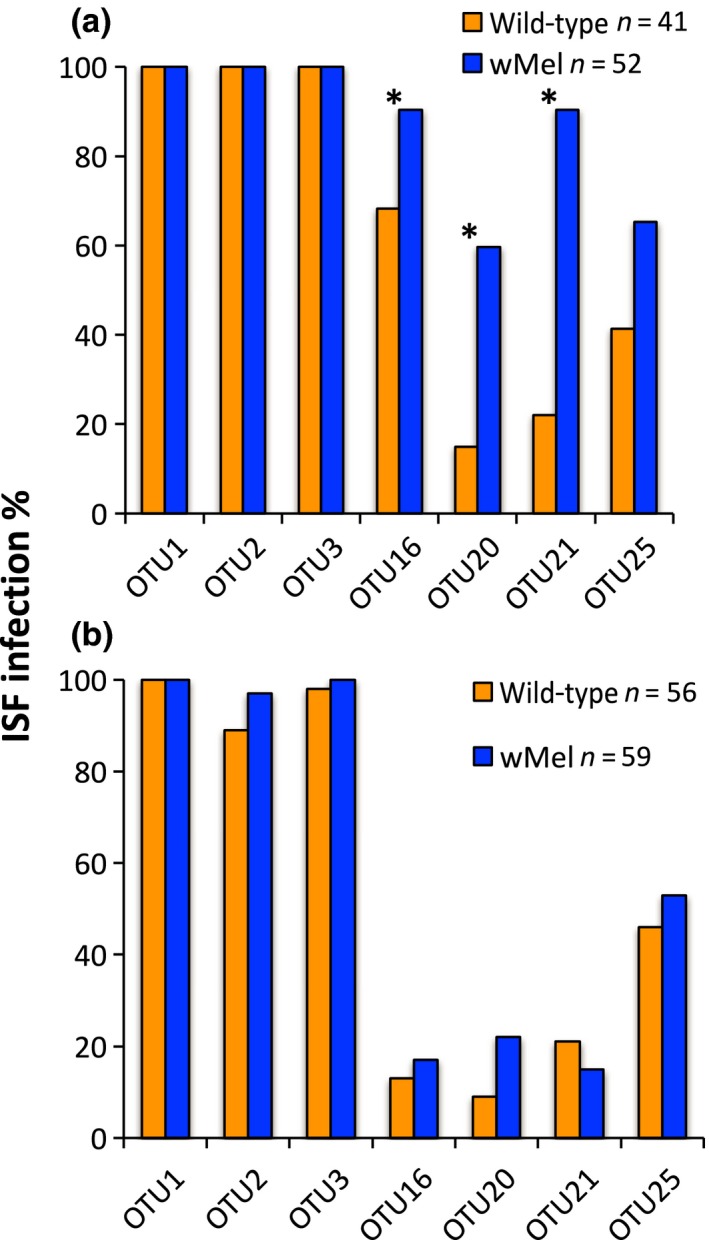
ISF infection rates in mosquitoes for a subset of OTUs as determined by RT‐qPCR. (a) In the field, three of the OTUs were more common in *w*Mel‐infected mosquitoes than WT (*p < .0125). (b) In the laboratory, there were no differences in ISF infection rates between *w*Mel and wild‐type mosquitoes in the laboratory (*p* = .27)

All but five OTUs (16, 20, 21, 23, and 25) were fixed in wild‐type and *w*Mel‐infected field populations based on sequence analysis. In the group of viruses not fixed, we found a significant effect of *Wolbachia* on infection frequency (Wald** **=** **5.49, *df* ****=** **1, *p* = .019), suggesting that *Wolbachia* was associated with higher rates of infection in the sequenced samples (Figure 2). We then tested whether these same trends were also present in the total set of samples (sequenced and not, *n* = 93) from the field using RT‐qPCR primers designed specifically for seven of the OTUs (Table 1). OTUs 1–3 were selected as they are closely related to well‐characterized ISFs (Figure 1, Table 2) and because they were fixed in both *w*Mel and wild‐type populations. Four additional OTUs (16, 20, 21, and 25) (Figure 2, Table 1) were selected given their differential distributions by sequencing. OTUs 1–3 were shown to be at 100% frequency (Figure 3a) in the larger set of field samples, recapitulating what was seen by sequence analysis. We then tested whether the frequencies of the remaining OTUs varied with respect to *Wolbachia* infection status and found there was a significant interaction between OTU and *Wolbachia* infection status (Wald** **=** **12.3, *df* ****=** **3, *p* = .006) and so proceeded with the four individual comparisons and a multiple test correction (revised α = 0.0125). OTUs 16 (Wald chi‐square** **=** **6.9, *df* ****=** **1, *p* = .009), 20 (18.3, *df* ****=** **1, *p* < .001), and 21 (41.0, *df* ****=** **1, *p* < .001) were significantly different, whereas OTU25 (5.26, *df* ****=** **1, *p* = .022) was not. In each case of significance, OTU frequencies were higher in *w*Mel mosquitoes (Figure 3a). OTUs 16 and 21 that were previously not found in the sequenced wild‐type samples were detected through RT‐qPCR. This may be due to the sensitivity cutoff employed with the sequence data whereby we excluded OTUs with <10 sequence reads. Lastly, we then determined if these differences were also seen in laboratory lines of *w*Mel‐infected and wild‐type mosquitoes. Unlike in the field, the rates of infection appear similar (Wald** **=** **1.177; *p* = .27) between the two lines (Figure 3b).

### 3.4 *Wolbachia* infection is associated with differences in abundance of ISFs

**Figure 4:**
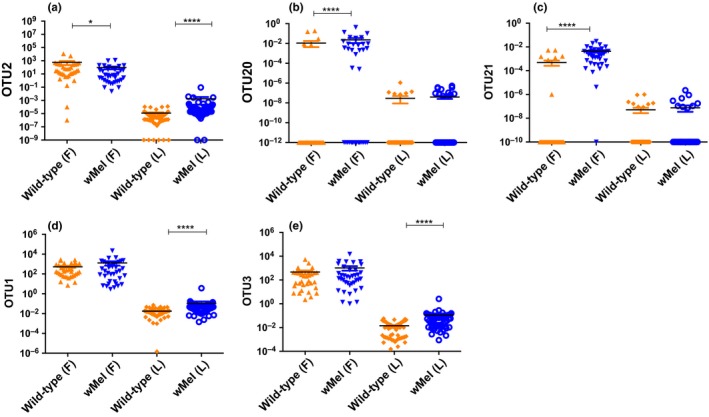
Relative abundance of identified ISF OTUs. (a) OTU2 decreased in abundance in *w*Mel mosquitoes in the field but increased in *w*Mel in the laboratory lines (b) OTU20 and (c) OTU21 were more abundant in *w*Mel mosquitoes in the field but were not different in the laboratory. (d) OTU1 and (e) OTU3 increased in abundance in *w*Mel laboratory mosquitoes only. *p < 05; ***p < .001; ****p < .0001

To determine whether the presence of *Wolbachia* had an effect on the abundance of ISFs, we compared the viral load of the seven selected OTUs between the wild‐type and *w*Mel mosquitoes under field and laboratory conditions by RT‐qPCR. Even though *Wolbachia* reduced the load of OTU2 in the field (Figure 4a), with the wild‐type having a higher load (*p* = .017) compared to *w*Mel mosquitoes, the opposite effect was observed in the laboratory where the *w*Mel mosquitoes had a significantly higher load (*p* < .0001) than that in the wild type. In the field, OTU20 (*p* = .0009) and OTU21 (*p* < .0001) were significantly more abundant in *w*Mel mosquitoes compared to the wild type (Figure 4b,c). This effect was not observed in the laboratory lines with no significant differences observed in loads of OTU20 (*p* = .17) and OTU21 (*p* = .91) between *w*Mel and wild‐type mosquitoes (Figure 4e,f). There were no significant differences between loads of OTU1 (*p* = .94) and OTU3 (*p* = .63) in *w*Mel and wild‐type mosquitoes in the field. In the laboratory lines however, *w*Mel mosquitoes consistently had a higher abundance of OTU1 (*p* < .0001) and OTU3 (*p* < .0001) compared to the wild type. In both the field and laboratory mosquito lines, there were no differences in the loads of OTU16 (*p* > .05) and the OTU25 (*p* > .05) between *Wolbachia*‐infected and wild‐type mosquitoes (Figure S1). In summary, regardless of the mosquito line, ISF loads varied considerably between field and laboratory environments with the former consistently harboring higher ISF density than the latter. This suggests that environmental conditions and differences in host genetic background may influence abundance of ISFs. Our findings demonstrate that in general, *Wolbachia* does not inhibit ISF loads in *Ae. aegypti* mosquitoes and in some cases may enhance them. It also suggests that the effect of *Wolbachia* on ISFs is virus‐specific and environmental conditions may influence this effect, such that the laboratory environment may not be predictive of the field.

## 4 DISCUSSION

Despite large‐scale field releases of *Wolbachia‐*infected *Ae. aegypti* mosquitoes (Hoffmann et al., 2011; McGraw & O'Neill, 2013; Ritchie, 2014), the antivirus or blocking effect of the symbiont on the naturally occurring ISFs in *Ae. aegypti* mosquitoes is currently not known. This work therefore examined whether *w*Mel *Wolbachia*‐infected mosquitoes sampled from field release sites in Australia and in laboratory populations exhibit symbiont‐associated changes in their ISFs. Generally, we found that in the field *w*Mel mosquitoes had both higher ISF infection rates and abundances compared to wild‐type mosquitoes. We should point out that the number of field populations tested is small and so the findings could be the result of environmental or stochastic factors. Mosquitoes from other field release sites around the globe should be profiled to determine whether these relationships are robust and generalizable. In the laboratory where *Ae. aegypti* are reared under optimal conditions, there was no difference in ISF infection rates between wild‐type and *w*Mel mosquitoes. However, *w*Mel mosquitoes did exhibit higher loads of ISFs compared to the wild type. These findings were unexpected given that *Wolbachia* has been extensively shown to inhibit flaviviruses of medical importance in *Ae. aegypti* (Bian et al., 2010; van den Hurk et al., 2012; Moreira et al., 2009; Walker et al., 2011).

A total of 26 OTUs were observed in the sequenced field samples with all mosquitoes harboring multiple, concurrent infections. Some of these OTUs were closely related to each other indicating they are likely variants of the same virus. Very few of the OTUs clustered with characterized ISFs present in the databases. This confirms observations in invertebrates (Shi et al., 2016), and more specifically in *Drosophila* (Webster et al., 2015), that there are novel insect‐specific viruses that are yet to be classified. Our study is limited by sequencing a short fragment (143 bp) that probably affects the ability to have unambiguous matches and could explain why some OTUs matched to more than one virus in the database.

ISFs were found to be ubiquitous in both the laboratory and field mosquito populations suggesting that these flaviviruses may be transmitted vertically. Studies by Bolling et al. (2012) and Lutomiah, Mwandawiro, Magambo, and Sang (2007) demonstrated that Culex flavivirus and Kamiti river virus are maintained in *Cx. pipiens* and *Ae. aegypti* mainly through vertical transmission with venereal transmission playing a minor role. Other studies carried out both under laboratory and field conditions further demonstrated that flaviviruses of medical importance including WNV (Baqar, Hayes, Murphy, & Watts, 1993), DENV (Bosio, Thomas, Grimstad, & Rai, 1992), and YFV (Beaty, Tesh, & Aitken, 1980) can be maintained in nature through vertical transmission.

We observed differences between field and laboratory ISF infection rates and loads with the field mosquitoes consistently having higher infection rate and abundance regardless of mosquito line. These differences between the two populations may be partly attributed to selection and founder effects (Lorenz, Beaty, Aitken, Wallis, & Tabachnick, 1980; Munstermann, 1994) as is common with laboratory colonies (Lorenz et al., 1980; Munstermann, 1980, 1994). There is also a possibility that environmental conditions in the field (Huber et al., 2002) predispose *Ae. aegypti* to increased ISF infection as factors such as temperature influence mosquito immunity and therefore mosquito–pathogen interactions (Huber et al., 2002; Murdock, Moller‐Jacobs, & Thomas, 2013; Murdock, Paaijmans, Cox‐Foster, Read, & Thomas, 2012; Murdock, Paaijmans, Bell, et al., 2012). High larval crowding (Alto, Lounibos, Mores, & Reiskind, 2008; Baqar, Hayes, & Ahmed, 1980), nutritional restrictions (Alto et al., 2008; Baqar et al., 1980; Grimstad & Haramis, 1984; Kho, Hugo, Lu, Smith, & Kay, 2016), and low temperature (Chambers & Klowden, 1990) have independently been shown to cause small body size with an accompanying increased susceptibility to arboviruses such as WNV (Baqar et al., 1980), DENV (Alto et al., 2008; Kho et al., 2016), and La Crosse virus (Grimstad & Haramis, 1984). Environmental variables therefore need to be tested empirically to establish their effects on ISF infection rates and load in *Ae. aegypti* mosquitoes. It is also possible that age could contribute to variation in ISF infection (Bolling et al., 2012), but the sampling of adults from wild populations did not allow for age control.

It was unexpected to observe a higher ISF infection rate in *w*Mel mosquitoes compared to the wild type in field *Ae. aegypti* populations. This sharply contrasts previous studies where *Wolbachia* infection significantly reduced the proportion of individuals infected with other flaviviruses such as DENV (Amuzu & McGraw, 2016; Amuzu, Simmons, & McGraw, 2015; Bian et al., 2013; Frentiu et al., 2014; Moreira et al., 2009; Walker et al., 2011), Zika (Aliota et al., 2016; Dutra et al., 2016), and YFV (van den Hurk et al., 2012). Our findings are, however, supported by studies performed in *Cx. tarsalis* and *An. gambiae* where the presence of *Wolbachia* increased the infection rate of WNV and *P. berghei*, respectively (Dodson et al., 2014; Hughes et al., 2012). In addition, *Wolbachia* has been observed to increase susceptibility of the DNA virus nucleopolyhedrovirus in the African armyworm, *S. exempta* (Graham et al., 2012). In laboratory lines, *Wolbachia* does not influence ISF infection rates suggesting that population genetic variation and differences in environmental conditions between the laboratory and field could be influencing *Wolbachia* interaction with ISFs. This hypothesis is not supported by Kho et al. (2016) and Caragata et al. (2013) who demonstrated that larval nutrition and adult carbohydrate intake did not affect DENV infection rates in *w*Mel mosquitoes. Temperature, in contrast, has been shown to determine whether the *w*AlbB *Wolbachia* strain inhibits, enhances, or has a neutral effect on oocyte infection rate and intensity of *Plasmodium yoelii* in *An. stephensi* (Murdock, Blanford, Hughes, Rasgon, & Thomas, 2014). Based on these studies and our findings, there is a need to further investigate the effect of environmental conditions on *Wolbachia*–ISF interactions in order to establish the role the environment may be playing in modulating ISF infection.

*Wolbachia* suppressed the abundance of OTU2, that is most similar to cell fusing agent virus, in the field populations and this is supported by other studies that found inhibition of this ISF by the *w*MelPop *Wolbachia* strain in *Ae. aegypti* cell lines (Schnettler, Sreenu, Mottram, & McFarlane, 2016; Zhang, Etebari, & Asgari, 2016). This suppressive effect, however, was lost in the laboratory where *Wolbachia* significantly enhanced loads of OTU2. Generally, we observed *w*Mel either enhanced loads of ISFs or had no significant effect, signifying that *Wolbachia* does not inhibit the success of these flaviviruses. The load of the insect‐specific virus Phasi Charoen‐like bunyavirus present in *Ae. aegypti* cells infected with the *w*MelPop *Wolbachia* was not shown to differ from those without the symbiont (Schnettler et al., 2016) thus supporting our observation that *Wolbachia* does not have an effect on ISF loads. Still, other studies have demonstrated pathogen enhancement by *Wolbachia* where the number of *Plasmodium relictum* oocytes that develop in the midgut of *Cx. pipiens* increased in the presence of the symbiont (Zele et al., 2014). This effect was also observed in *An. gambiae* where *w*AlbB *Wolbachia* strain significantly increased oocytes levels of *P. berghei* (Hughes et al., 2012). The fact that *Wolbachia* did not have a significant effect on loads of OTU16 and OTU25 suggests that the effect of *Wolbachia* on ISF are virus‐specific. This supports other studies in which contrasting results were observed for closely related species where the *w*AlbB *Wolbachia* strain enhanced *P. berghei* (Hughes et al., 2012) but inhibits *P. falciparum* (Hughes, Koga, Xue, Fukatsu, & Rasgon, 2011) in *An. gambiae*.

The effect of the *Wolbachia–*ISF relationship on viruses of medical importance has not been examined in mosquitoes. It is possible that ISF enhancement by *Wolbachia* may not have an effect on arboviruses (Crockett et al., 2012; Kent et al., 2010). Alternatively, it could lead to inhibition of arboviruses as was observed in the case of WNV and Murray Valley encephalitis (Bolling et al., 2012; Hobson‐Peters et al., 2013), further strengthening the pathogen blocking effect of *Wolbachia*. Given that ISF is common and widely distributed in *Ae. aegypti* mosquitoes, this could be advantageous to the current *Wolbachia*‐dengue control strategy. More concerning is the possibility of ISF enhancement resulting in increased susceptibility of mosquitoes for arboviruses as was reported in a different study for WNV (Newman et al., 2011). This would have serious consequences for the current *Wolbachia*–DENV control strategy as *Wolbachia*‐infected mosquitoes will facilitate arbovirus proliferation instead of limiting them. Finally, our findings point to the need to carefully examine environmental conditions before embarking on *Wolbachia–Ae. aegypti* field releases as the *Wolbachia*‐pathogen effects observed in the laboratory may not be representative of the field.

## CONFLICT OF INTEREST

None declared.

## DATA ACCESSIBILITY

All data are available at Figshare https://doi.org/10.4225/03/5aa1a8b0af9f3.

## AUTHOR CONTRIBUTIONS

EAM conceptualized and supervised the project. CK provided field samples. HEA did the experimental work. KT, HEA, EAM, RH, and DP analyzed data. The manuscript was written by EAM and HEA.
