# Supplementary figures and images for: Wolbachia enhances insect‐specific flavivirus infection in Aedes aegypti mosquitoes
Source: Ecol Evol. 2018 May 8;8(11):5441–54. doi: 10.1002/ece3.4066 (PMC6010864; doi:10.1002/ece3.4066)

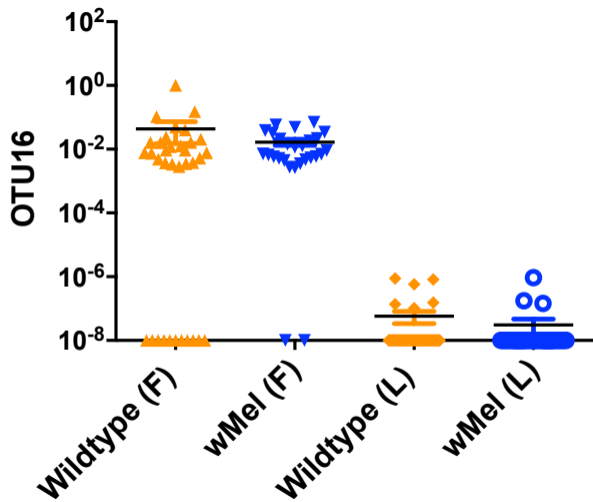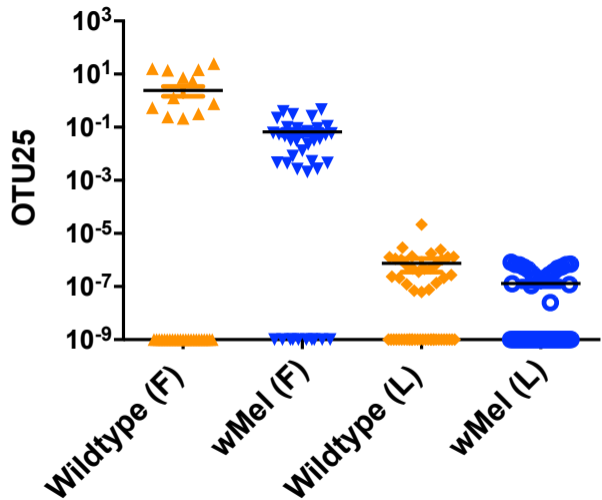

Supplement: Supplementary file 1 [file ECE3-8-5441-s001.pdf]
