# Supplementary material for: Wolbachia enhances insect‐specific flavivirus infection in Aedes aegypti mosquitoes
Source: Ecol Evol. 2018 May 8;8(11):5441–54. doi: 10.1002/ece3.4066 (PMC6010864; doi:10.1002/ece3.4066)
Supplement: Supplementary file 2 [file ECE3-8-5441-s002.docx]

# Supplementary Table 1: Operational taxonomic units (OTUs) and their sequences

| **OTU** | **Sequences** |
| --- | --- |
| 1 | GAAAAAAGAGAAGCAACCGACCATAGCTGGGGGGCCGAAAGGATCCCGGACCATTTGGTATATGTGGCTGGGAAGTCGATATCTGGAATATGAGGCCTTG |
| 2 | CAAGAAGGAGAAAAAGCCCAGCCTAGCTGGTGAGGCCAAGGGCTCAAGGACCATCTGGTATATGTGGCTTGGGAGCCGGTTCCTTGAATTTGAGGCTCTA |
| 3 | GAAAAAAGAAAAGAAACCAACCATAGCTGGGGAGCCGAAAGGATCCCGGACCATTTGGTATATGTGGCTGGGAAGTTGATATCTGGAATATGAGGCCTTG |
| 4 | GCTTCCTCAACGCGGACCATTGGAAGCATTAACCTCGCATGTACATCATCATGGGGCTTCCTCAATGCAGACCACTGGCT |
| 5 | GCTTTCTCAACAAGGACCACTGGAAGCATTAACCTCGCATGTATAACATGATGGGGCTTCCTCAATACAGACCACTGGTT |
| 6 | GCTTCCTCAACGCAGACCACTGGAAGCATTAACCTCGTATGTATATCACGATGGGGCTTTCTCAATAAGGACCATTGGAGG |
| 7 | CATGTGTGTAAACATGATGGGGCTTCCTCAACGAGGACCACTGGAAGCATTAACCTCGGTGTACATCATGATGGGGCTT |
| 8 | GCTTTCTCAACGCGGACCATTGGAAGCATTAACCTCGCATGTACAACATGATGGGGCTTTCTCAATAAGGACCACTGGCC |
| 9 | GCTTCCTCAACAAAGACCACTGGAAGCATTAACCTCGTATGCATAACATGATGGGGCTTTCTCAATGAGGACCATTGGCC |
| 10 | CGATGTGTGTATAACATGATGGGGCTTCCTCAATACAGACCACTGGAAGCATTAACCTCGTGTGCACATCATCATGGGCTTCCTCAATAAAGACCACTGGAC |
| 11 | TACGTGTGTATATATGATGGGGCTTTCTCAACGAAGACCATTGGAAGCATTAACCTCGCGTGTATATCACCATGGGCTTCCTCAATAAAGACCACTGGGC |
| 12 | CAGGCTTCCTCAATACGGACCACTGGGAGCATTAACCTCGTGTGTATAACATGATGGGGCTTCCTCAACAAAGACCATTGGCC |
| 13 | GAAAAAAGAGAAGCAACCGACCATAGCTGGGTGGCCGATGACATCGCCGGCTGGGAAGTCGATATCTGGAATATGAGGCC |
| 14 | GCTTTCTCAACGAGGACCATTGGAAGCATTAACCTCGTGTGTATATCACGATGGGGCTTCCTCAACAAAGACCATTGGCT |
| 15 | GCTACCTCAATGCGGACCACTGGAAGCATTAACCTCGTGTGCACAATATGATGGGGCTATCTCAACGAGGACCACTGGAAGCATTAACCTTGCGTGTACATCATGATGGGGCTTCCTCAACAAGGACCATTGGCC |
| 16 | AGACGTGTGCACAACATGATGGGGCTTTCTCAACACGGACCATTGGGCTTCCTCAATAAGGACCATTGGCC |
| 17 | GCTTTCTCAACACGGACCATTGGAAGCATTAACCGCGTGCACATCATCATGGGGCTTTCTCAACGAAGACCATTGGTG |
| 18 | GCCAATGGTCTTTATTGAGAAAGCCCCATGGTGATGTGCATGCGAGGTTAATGCTTCCAGTGGTCCGTGTTGAGGAAGCCCATCATGTTATACACACA |
| 19 | GCTCCCTCAATGCAGACCACTGGAAGCATTAACCTCGCATGCACATCATGATGGGGCTCTCAACAAAGACCATTGGAG |
| 20 | CTTTCTCAACACGGACCACTGGAAGCATTAGCCGCGTGTATAACACCATGGGCTTTCTCAACACAGACCATTGGACG |
| 21 | GCTTCCTCAACACGGACCAGTGGAAGCATTAACCTTGCGTTACAACATGATGGGGCTTCTCTACAAGACCACTGGCA |
| 22 | GCTTCCTCAATGAGGACCACTGGAAGCATTAACCTCGTGTGTATATCATGATGGGGCTTCCTCAATAAAGACCACTGGCC |
| 23 | CAGGCTTCCCCAACAAAGACCACTGGAGCCTGTGTGTATACCATGATGGGGCTTTCTCAACACAGACCACTGGCTT |
| 24 | GCTTCCTCAACGAGGACCATTGGAAGCATTAACCTCGCATGTACATCATCATGGGCTTTCTCAACACAGACCACTGGGG |
| 25 | CTTCCTCAATACAGGCCACTGGGAGCATTAACCTTGTATGCACATCATCATGGGGCTTTCTAACACGGACCATTGGC |
| 26 | CTTACTCAACACGGACCACTGGAAGCATTAACCTCGTGTGCATATCATGATGGGGCTTCTCAATACAGACCACTGGCG |
